# A Strange Mosaic-Like Skin Pigmentation in a 2-Year-Old Child

**DOI:** 10.5826/dpc.1104a135

**Published:** 2021-10-01

**Authors:** Linda Tognetti, Davide Cosetti, Pietro Rubegni

**Affiliations:** 1 Dermatologic Sciences Unit, Department of Clinical Medicine and Immunologic Sciences, University of Siena, Siena, Italy

## Case Presentation

A 2-year-old boy presented to the first aid for a strange skin pigmentation present since 2 months. Personal history was negative for application of perfumes, new clothes, drug intake, sun-exposure, or any change in usual habits or alimentation. There was no history of atopy, hypersensitivity, skin reaction to insect bites, or any other dermatosis. The lesions were asymptomatic. Dermatologic consultation was required.

## Teaching Point

**Figure 1:**
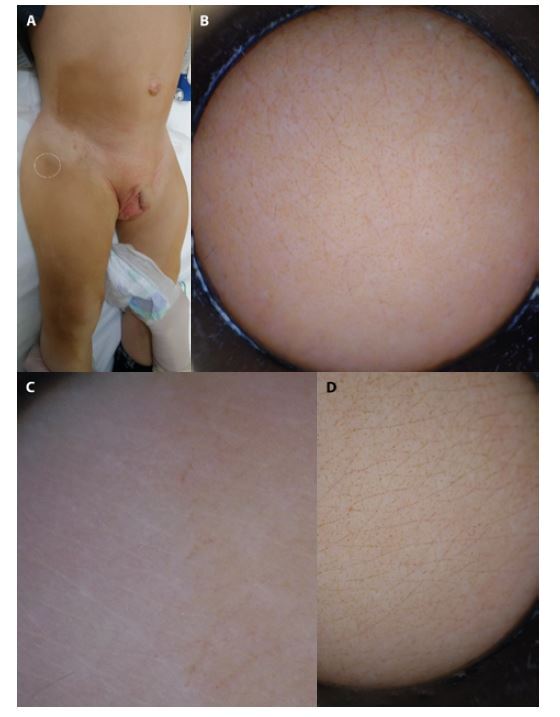
(A) Brownish pigmented areas with overall well-defined margins on the lower abdomen, and the proximal legs. (B) Polarized dermoscopy performed over the right external tight on the hyper pigmented area, of the abdomen (C) and of the knee area (D) revealing the presence of an exogenous pigmentation, limited to the upper stratum corneum, sparing adnexa follicular openings.

Brownish pigmented areas with overall well-defined margins were present on the lower abdomen, the anterior surface of the thighs, and the proximal legs (Figure 1A). Polarized dermoscopy highlighted a light-brown pigment mesh localized to dermatoglyphics (Figure 1, B–D).

Clinical and dermoscopic findings were consistent with an exogenous pigmentation diagnosis [1]. Clinical history investigation finally revealed the use of a specific type of cleaning wipe for babies used for the child skin cleansing. Recently, that specific cleaning wipe was reported to cause orangish-to-browhinsh hyperpigmentation due to alteration of ascorbic acid during packaging [2,3].
